# Curricular response to COVID-19: real-time interactive telehealth experience (RITE) program

**DOI:** 10.1080/10872981.2021.1918609

**Published:** 2021-04-22

**Authors:** Joseph E. Safdieh, Jennifer I. Lee, Lona Prasad, Mary Mulcare, Brian Eiss, Yoon Kang

**Affiliations:** a Gertrude Feil Associate Dean for Curricular Affairs and Professor, Department of Neurology, Weill Cornell Medicine, New York, NY, USA; b Associate Professor, Weill Department of Medicine, Weill Cornell Medicine, New York, NY, USA; c Associate Professor, Department of Medicine, Weill Cornell Medicine, New York, NY, USA; d Senior Associate Dean (Education) and Richard P. Cohen Associate Professor, Weill Department of Medicine, Weill Cornell Medicine, New York, NY, USA

**Keywords:** Clerkships, telehealth, covid-19, clinical curriculum

## Abstract

**Introduction**. The COVID-19 pandemic placed an unprecedented strain on academic healthcare systems necessitating a pause in medical student teaching in clinical care settings, including at Weill Cornell Medicine (WCM). WCM had a preexisting telemedicine curriculum, but limited opportunities for students to apply knowledge and skills related to direct virtual patient care. The authors describe the rapid implementation of real-time interactive telehealth experience (RITE) courses for clerkship students to allow for meaningful engagement in remote patient care and continuation of academic progress during the pause.

**Methods of Course Development**. Medical school administration disseminated a request for proposals for RITE courses conforming to the WCM electives format with rapid turnaround time of 1 week or less. Requirements included remote care activities, goals and objectives, general logistics, supervision methods and standards of achievement. RITE courses were developed in outpatient medicine, inpatient medicine, psychiatry and women’s health. A lottery process was developed to register students for the approved courses.

**Course Implementation and Evaluation**. Using the technical platform and standard course registration process, students were assigned to 74 of 76 available RITE course slots. Students participated in supervised remote direct patient care and also provided critical support for frontline healthcare workers by performing remote clinical tasks. Online teaching and reflection sessions were incorporated into each RITE curricular offering. Student feedback was overall positive ranging from 3.33–4.57 out of 5.

**Discussion**. The COVID-19 pandemic created a need to rapidly incorporate telehealth models in order to continue to deliver patient care and an opportunity to develop innovative remote educational experiences. We developed a framework for structured real-time interactive telehealth experiences to address COVID-19 related curricular needs that will be continued post-COVID-19. This expanded telehealth curriculum for our students will provide standardized training in telehealth logistics, communication techniques, and care delivery now essential for graduating medical students.

## Introduction

The impact of the COVID-19 pandemic on healthcare systems in the USA has been unprecedented. New York City quickly became an epicenter of disease activity in the late winter and early spring of 2020, leading to a rapidly increasing number of critically ill patients admitted to academic medical centers^1^. This surge of patients strained healthcare resources and necessitated increased capacity in intensive care unit beds and redeployment of residents and teaching faculty^2^. Personal protective equipment (PPE) shortages, limited availability of testing, and multiple residents and faculty out on self-isolation or quarantine further impacted the strain on the system^3^. Additionally, for the first time in the history of our general medicine services, patients were placed in complete isolation from social support, forced to face the fear of potentially life-threatening diagnoses alone. In light of all of these factors, the clinical care setting was no longer an effective or sustainable teaching and learning environment for medical students.

In early March 2020, in consultation with its primary teaching hospital system, Weill Cornell Medicine (WCM) made the difficult but necessary decision to suspend all medical student teaching in clinical care spaces. This decision was made by multiple other medical schools and supported by a statement from the Association of American Medical Colleges^4^. All clerkships underway at the time of the pause quickly transitioned to a remote learning format for the next 2 weeks using recorded lectures, interactive remote cases, and live-streamed discussions and readings to allow students to complete these courses. When these clerkships ended the last week of March 2020, the challenge was to develop an approach to continuous academic progress for the clinical clerks during the pause of in-person clinical training.

Central medical education and clinical curriculum leadership agreed that core clerkships could not be continued in the absence of patient contact since the centerpiece of clerkship training in medical school is direct patient care. Additionally, person power for core educational activities was strained as many clerkship teaching faculty and clerkship directors were deployed for COVID-19 related clinical needs. The challenge for second-year clinical clerks was acute since they had only completed a quarter of their core clerkship year. These students were in a position where graduation timelines would be jeopardized without ongoing academic progress.

## Methods of course development

In response to the rapidly evolving COVID-19 pandemic and its impact on the education mission, the Senior Associate Dean of Education convened a COVID-19 response steering committee (CRSC) which met daily throughout the months of March and April 2020. The following guiding principles for the medical education program were established by the CRSC [1]; prioritizing public health imperatives and the safety of students, faculty, staff, and patients [2], continuing delivery of effective content to meet core medical education goals and objectives [3], continuing attention to student academic support, advising, and wellbeing programming, and [4] exercising flexibility of thought and approach, in alignment with any relevant accreditation and state guidelines to allow for adjustments in response to changes in the pandemic and for potential innovation. CRSC members were assigned to individual COVID-19 response committees (CRCs). Each CRC was charged with developing strategic approaches to an assigned area of the medical education program (e.g., pre-clinical curriculum, clinical curriculum, student advising and support), with adherence to guiding principles.

The CRC: Clinical Curriculum was tasked with development of strategies to ensure students would continue making effective academic progress in the clinical curriculum. This CRC recognized that telemedicine was both a necessity during COVID and a well-established area of institutional strength, to which students previously had limited exposure in the patient care setting. Therefore, a recommendation was made to capitalize on this strength and leverage telemedicine to enable academic progress and meaningful student engagement in the clinical care mission during COVID-19.

In response to this recommendation, the clinical curriculum office put out a request for proposals to clinical department educational leaders for real-time interactive telehealth experiences (RITE) with a five-day response deadline. The experiences would include supervised and educational task-oriented responsibilities to enhance remote clinical care efforts. The goal was to design a menu of credit-bearing options in which students could enroll over a projected 8-week pause in on-site clinical care activities. Faculty were asked to follow the standard WCM elective format and list goals and objectives, prerequisites, course length, maximum enrollment, general logistics, supervision methods and standards of achievement. In addition to patient care responsibilities, online didactic teaching in addition to debrief and reflection sessions were required. All RITE courses were graded pass/fail, in alignment with all elective offerings at our institution.

Four departmental groups responded with RITE proposals. These included faculty from ambulatory and emergency medicine (EM), obstetrics and gynecology, psychiatry and hospitalist medicine (Table 1). The proposals were initially vetted by the Assistant Dean for Clinical Curriculum and then underwent expedited review and approval by the Director of Electives. After approval, the offerings were given course codes and entered into the registration system. Each RITE course was 4 weeks in length and offered two consecutive times, allowing students to potentially register for 2 different courses over the 8-week pause in on-site clinical care activities. The capacity across the RITE courses was 38 students per four-week block, for a total of 76 course slots over the 8-week pause. An online class meeting was conducted to notify eligible students about the RITE offerings, the process by which they could enter preferences and be enrolled. Students were registered using the same online lottery course registration software (OASIS, Madison WI) and process used for registering students to clerkship blocks. Students were asked to enter their choices for RITE courses ranked in order of preference by a set deadline and the lottery system then used a random number generator to assign students to available slots based on their preference for course and month. The lottery process was completely computer driven and no faculty or administrative staff were able to pre-assign any outcomes.

**Table 1.** Real-time interactive telehealth experiences (RITE)

Name of RITE	Sponsoring Department(s)	Length	Max capacity of students per block (total actual enrollment over 8 weeks)
Frontline Care: Virtual Team Member for COVID hospital medicine teams	Medicine	4 weeks	15 (28)
Telehealth in the Age of COVID-19	Medicine, Emergency Medicine	4 weeks	15 (30)
Telehealth for Women’s Care	Obstetrics and Gynecology, Reproductive Endocrinology	4 weeks	5 (10)
Psychiatry Liaison Telehealth Experience	Psychiatry	4 weeks	3 (6)

## Course implementation and evaluation

Sixty students opted to register for RITE program offerings and were entered into the registration lottery. Of the 76 available slots, 74 were filled in the lottery process by 60 unique students. Analysis of the lottery results revealed that 89.7% of students received their top choice, and 100% received 1 or more courses in their top three. The RITE programs that were developed spanned various specialties of medicine. All the offerings incorporated remote patient care experiences, but each had an individualized general structure and learning objectives (Table 2).

**Table 2.** Summary of learning objectives in the RITE courses

Name of RITE	Learning objectives
Frontline Care: Virtual Team Member for COVID hospital medicine teams	Actively engage in frontline care for sick and critically patients as a core member of the Hospital Medicine COVID team Obtain valuable patient related information to the team such as HPI, past medical histories, home medication histories Participate in goals of care discussions led by the attending hospitalist with patients and their families Identify various safety and systems issues highlighted through the care of COVID19 patients
Telehealth in the Age of COVID-19	Demonstrate clinical skills in history taking and the physical assessment of patients with confirmed or suspected COVID-19 utilizing telephone and/or video technology. Provide follow up medical advice and counseling for patients with suspected or diagnosed COVID-19 in various clinical settings and appropriately recognize patients in need of immediate referral, facilitating their return to care. Integrate into and communicate within a multidisciplinary team of healthcare professionals utilizing telehealth technology to provide and document patient care remotely. Reflect on the service-oriented nature and clinical and emotional challenges of caring for patients via telehealth in the midst of the COVID-19 pandemic. Collaborate using peer support to reflect and troubleshoot obstacles in the context of telehealth communication.
Telehealth for Women’s Care	Participate in high risk obstetrics telemedicine visits and postpartum telemedicine visits Describe adverse outcomes associated with hypertension and diabetes mellitus in pregnancy List the criteria for achieving optimal glycemic control during pregnancy Describe normal and abnormal blood pressures in pregnancy, diagnostic criteria for hypertensive diagnosis of pregnancy and the use of anti-hypertensive medications in pregnancy Understand components of routine postpartum care Describe the benefits of breastfeeding and common challenges in initiation and maintenance of breastfeeding Describe postpartum contraception options
Psychiatry Liaison Telehealth Elective	Summarize how a liaison service is constructed and operated Describe basics of statistics and logistics of building and maintaining a database focused on public health outcomes Describe how to optimize online resource availability to support clinicians and staff Conduct ongoing literature search of one or more areas of research in liaison/crisis response teams.

### Description of individual RITE Programs

#### Frontline care: virtual team member for COVID hospital medicine teams

Patient Care Related Activities

The Frontline Care: Virtual Team Member for COVID Hospital Medicine Teams course was developed to address one of the greatest needs identified within our institution during this unprecedented crisis – providing social support to an increasingly vulnerable COVID-19 suspected and positive patient population. Students were assigned to hospital medicine teams and were responsible for maintaining virtual contact with persons-under-investigation (PUI) and COVID-19 positive patients during a time when no visitors were allowed in the hospital.

Students were available to function as virtual team members (VTM) Monday through Friday and assigned up to five patients at a time. Students were directly supervised by a hospitalist attending physician. Patients were excluded if they declined follow-up calls, were unable to engage in conversation due to cognitive impairment or oxygen requirements, or were admitted to the intensive care unit. Students used phone interpreters to communicate with limited English proficiency patients. As the number of post-COVID-19 recovery patients remaining in the hospital increased, students were also assigned to the COVID −19 Recovery Unit teams for the latter portion of the rotations.

VTMs were primarily responsible for providing daily updates by phone and video calls to isolated patients and family members emphasizing essential communication skills and tasks related to care coordination. Clinical tasks were performed based on need and included obtaining and updating outpatient medication histories for new admissions, obtaining collateral information from the primary outpatient physician or other source, performing verification of insurance coverage and availability of high-risk discharge medications, providing discharge instructions to both patients and their families regarding post-COVID-19 care and management, and reviewing checklists to help patients prepare for discharge follow-up telehealth visits.

Students were also directed to call patients on their hospital room landline or cell phone, including video calls when available, with a goal of twice-daily check-ins arranged at the patient’s convenience as an ‘appointment’ to help orient them to time. Students were clearly instructed to communicate any change in status or new issues raised by the patients or recognized by the students during the daily calls to the primary medical team. Students documented the calls in the electronic medical record and this documentation was routed to their attending supervisor for review and approval.

Didactic Activities

Faculty experts in infectious disease, mental health, medical ethics, quality improvement and patient safety conducted mandatory weekly didactic teaching sessions through video conferences throughout the course. Students were also divided into two groups of their choice to participate in either a mini-root cause analysis of a medical error they identified during the course or a quality improvement project to improve and standardize patient handoffs during a time of rapid care transfers across settings.

Student Feedback and Assessment

During weekly debrief sessions, students and attendings shared experiences and discussed challenges and barriers in order to assess and adjust the course content and structure in real time.

Students were assessed by faculty and house staff using the standard WCM clinical student performance evaluation form. The form includes questions on medical knowledge, clinical reasoning, history taking, physical exam performance, presentation skills, teamwork skills and overall professional and ethical behavior. Evaluators also provided qualitative comments, which were compiled by the course director to synthesize a narrative evaluation. Students successfully completed the course if the average summary score of their composite evaluations was above the WCM passing threshold. Final grades were reported as Pass/Fail,

### Telehealth in the age of COVID-19

Patient Care Related Activities

The Telehealth in the Age of COVID-19 course engaged students in the follow-up care of patients with confirmed or suspected COVID-19 after discharge from the emergency department or outpatient clinics. As part of the course, students also participated in longitudinal care of homebound patients with increased mental health needs and isolation in the context of the COVID-19 pandemic. The course was a completely remote and supervised experience, allowing students to be actively involved in clinical care even at the height of the COVID-19 pandemic, doing so in a safe learning environment. Students worked with faculty at two out of the three ‘virtual’ clinical sites: primary care, emergency medicine and the Center on Aging.

At the primary care and emergency medicine sites, students contacted recently evaluated confirmed or suspected COVID-19 patients listed in a HIPAA-compliant database. Students were trained in and used a scripted electronic medical record documentation template to facilitate the encounters. They also used a decision tree to help determine the next steps during and after the encounters. Students had site-specific faculty preceptors who discussed cases in real-time if there was any indication that there was a change in patient clinical status, or the patient was doing poorly at home. Students were responsible for documenting and conveying the outcome of each call to the faculty member assigned as their preceptor for the day as well as to the patient’s primary outpatient provider. Faculty preceptors reviewed all phone call notes during each session. The students also had two, 3-hour telehealth attending shadow shifts, where they joined emergency medicine attending physicians doing telehealth encounters via video conferencing software.

Students were responsible for counseling patients and providing them with ongoing anticipatory guidance, with connecting them back to their primary providers as they recovered, and in cases where urgent re-evaluation was recommended after discussion with a preceptor, students were also responsible for conveying these plans to patients. In many cases, particularly at the primary care site, this meant that students had a ‘panel’ of patients that they followed for days or even weeks, leading to opportunities for rapport building and continuity with patients and their families.

At the Center on Aging site, students collaborated with social workers to create a ‘telephone reassurance’ program where students also followed panels of patients identified by their providers at the practice as vulnerable due to their chronic conditions or social isolation. They utilized a specific telephone script to make follow-up calls anywhere between 1 and 3 times a week to patients, depending on a patient’s needs. They documented all calls and forwarded them to their primary providers at the practice and to the group’s geriatrics social worker. She and one of the course directors discussed cases in real-time if the patient or students had any concerns about a patient’s safety at home. Students were trained to identify and refer patients to the practice’s social worker or directly to community organizations if they discovered issues of food scarcity, social isolation or medication access. Students were trained in and performed telephonic mental health screens and referred many patients to the mental health team at the Center on Aging.

Didactic Activities

On orientation day, the students completed ‘Introduction to Telemedicine’, a virtual 1-hour course created for all providers doing telehealth visits at our institution. Course faculty conducted didactic sessions on management of COVID-19 and on telemedicine techniques. Students also participated in observed simulated telephone follow-up calls. In addition, students participated in reflective exercises facilitated by faculty members who highlighted the service-oriented learning innate to the clinical work they undertook during this elective experience. At these sessions, there was also group debriefing on student experiences and feelings as ‘providers’ during the pandemic.

Student Feedback and Assessment

Each student received regular real-time feedback from faculty preceptors on their medical knowledge, communication skills and their abilities at clinical reasoning and differential diagnosis based on their clinical presentations and in some cases, direct observation of their telehealth encounters. This included direct observation and feedback on clinical skills through joint phone calls with supervising clinicians.

Student performance was assessed by faculty preceptors as well as social work and nursing colleagues using the standardized WCM elective student performance evaluation form. This form included Likert scales to measure core clinical competencies in multiple domains, including professionalism, interprofessional communication and teamwork. Students passed if the average summary score of their composite evaluations was above the WCM passing threshold. Overall grades were pass/fail.

### Telehealth for women’s care

Patient Care Related Activities

The Telehealth for Women’s Care course integrated students remotely into the general gynecology, routine and high-risk obstetrical (HROB) and postpartum outpatient clinical care teams. Students were granted access to provider scheduling templates through the electronic medical record system and took part in video visits with all providers. Students were instructed to email the providers to whom they were assigned on the day before each telehealth session. This allowed the students ample time to review the patient charts, ask relevant background questions and prepare for their involvement in the upcoming visits with the provider and patients. As the students’ knowledge base and comfort level with the technology and clinical concepts increased, they became more actively engaged with patient interactions. Students participated in the video visits by eliciting histories and discussing management plans under supervision by faculty, residents and fellows.

All students rotated for 1 week with the high-risk obstetrical (HROB) faculty monitoring and caring for pregnant patients, specifically with hypertension and diabetes mellitus. During this week, students learned about the diagnostic criteria for hypertension and diabetes mellitus in pregnancy, the associated adverse outcomes in both, and treatment interventions that may safely be instituted in pregnancy.

Students rotated for 2 weeks engaging in postpartum telehealth video visits. During these visits, they addressed patient concerns regarding post-delivery care, including breastfeeding, resumption of sexual activity and use of contraception. Under supervision, they provided postpartum counseling to patients on sensitive issues associated with breastfeeding and family planning. Students also took part in other routine obstetrical and gynecological problem-focused visits as they occurred during these 2 weeks. They had the opportunity to participate in counseling sessions with the social workers as well.

Didactic Activities

The HROB week included three hours of interactive live online teaching with the HROB faculty. During the 2 post-partum care and general gynecology weeks, students participated in interactive teaching sessions with the course director on the topics of lactation, contraception, prenatal care and pregnancy during COVID-19. During the last week students participated remotely in educational sessions and in academic debate regarding the administration of infertility treatments during the COVID-19 pandemic. These sessions were led by a faculty member from the reproductive endocrinology and infertility (REI) department. The education sessions covered an overview of assistive reproductive technologies (ART), methods of in-vitro fertilization and specific challenges surrounding ART arising during the COVID-19 pandemic. Students were then divided into two groups, and the groups were assigned to either advocate for or against using ART during COVID-19. Students carried out independent research about the topic and then debated the pros and cons.

In addition to the formal didactic curriculum, the course director met with the students daily. These sessions consisted of review of challenging topics the students were seeing, course-specific concerns and feedback, future research and case presentations. Presentation topics were both COVID-19 and non-COVID-19 related. Future research ideas incorporating quality improvement, mental health, healthcare equity and literacy as well as ideas for biomedical innovations were discussed. The opportunity for student involvement in creating research protocols for both group and individual projects was established.

Student Feedback and Assessment

Students were evaluated on their level of participation, knowledge base, patient interaction and communications skills. Each student was required to complete two formal write-ups; either a clinical case of their choice encountered during their high-risk obstetrical week or during their general ob-gyn/postpartum week, as well as one topic associated with infertility. For the clinical write-up of either the high-risk obstetrical or general ob-gyn/postpartum case, students were required to include the following: complete history and physical exam, assessment and plan with a problem list and a 2–3 page discussion of one of the problems with a brief review of the basic science related to the condition with citation of relevant sources from the literature when applicable. A different format for the REI write-up was implemented since this assessment was not based on direct patient interactions. Students were expected to provide an overview of management options in addition to a discussion of ethical concerns and psychosocial issues associated with either providing or withholding IVF treatment during the COVID pandemic. To calculate the overall grade, observed communication and interaction with patients on the phone and during tele-health visits accounted for 50%, each formal case write-up accounted for 20%, and 10% was based on level of preparedness and participation during the group sessions with the course director. Final grade was reported as pass/fail.

### Telehealth psychiatry liaison elective

Patient Care Related Activities

The Telehealth Psychiatry Liaison Elective incorporated students into a newly formed psychiatry liaison service in response to COVID-19. The service assigned liaisons from psychiatry to clinical departments and programs throughout the hospital. Liaisons offered virtual group town hall meetings and debriefing sessions, responsible for developing and implementing tailored interventions. As part of the course, students assisted in conducting the department town halls with primary oversight by an attending psychiatrist or psychologist. To ensure that students were not providing psychiatry liaison services to potential future evaluators, they only participated in town halls for nonacademic staff. They also observed training meetings for liaisons on crisis intervention.

This course also included a research component as the students were also required to participate in building and managing an outcomes-focused database for the treatment team. They also assisted the overall effort of the psychiatry liaison service by performing literature reviews in preparation for grant submission on topics such as posttraumatic stress disorder and moral injury as a result of COVID-19.

Didactic Activities

For the first few days of the course, students learned the principles of forming a consult psychiatry liaison service. Students then learned how to triage requests from departments to develop group town halls. They were also trained in research database design.

Student Feedback and Assessment

Students were assessed based on a number of equally weighted components. These components included [1] degree of preparation for and participation in the didactic sessions [2], degree of preparation for the town halls [3], overall adherence to WCM student professionalism behaviors, and [4] the quality of their literature reviews and database management. Assessment was performed by faculty members who interacted with the students and the course director collected feedback from faculty about student performance. In order to pass the elective, students were required to meet or exceed minimum expected performance standards in each of the four domains of evaluation.

### Student assessment

Students were assessed using the standard WCM elective student performance evaluation form. The form was completed for each student by the course director within 4 weeks of the end date of the course. The form includes questions on medical knowledge, clinical reasoning, history taking, physical exam performance, presentation skills, teamwork skills and overall professional and ethical behavior. Course directors were asked to specifically assess how students performed these tasks using a telemedicine approach. Course directors were also asked to include narrative comments, which they collected from the residents and faculty supervisors who worked with the students. RITE course grading was pass/fail, and all students passed the RITE courses.

### RITE program evaluation

Students were asked to evaluate the RITE programs using the standard WCM course evaluation form, with one custom question added: ‘Rate the added value of this course as a new learning experience, above and beyond what was learned in the required curriculum.’ Students generally rated the RITE programs highly, with a range from 3.33 to 4.67 out of 5 possible points in important categories including course quality, course organization, and amount of patient care responsibilities (Table 3). The courses with the highest ratings were those in which students were given more direct patient care activities and engaged in a supervised manner with patients via telemedicine.

**Table 3.** RITE Program Evaluation Scores (range 1–5)

Name of RITE	Total number of evaluations submitted	Overall Course Quality	Amount of Patient Care Responsibility	Value of Course as a New Learning Experience	Overall Organization
Frontline Care: Virtual Team Member for COVID hospital medicine teams	26	3.50	3.55	3.64	3.59
Telehealth in the Age of COVID-19	28	4.57	4.30	4.57	4.30
Telehealth for Women’s Care	8	4.00	4.00	4.38	3.88
Psychiatry Liaison Telehealth Elective	6	3.67	3.67	3.33	3.33

Students provided narrative comments related to perceived strengths and areas of improvement for the RITE program courses. Themes that emerged in a summary of program strengths included [1] ability to feel ‘useful’ to both patients and front-line providers despite being on a clinical curriculum pause [2], applied telemedicine experience, and [3] development of longitudinal relationships with patients. Themes that emerged in a summary of areas to improve included [1] challenges inherent in telehealth encounters that occurred only via telephone and [2] experiences that were more observational than experiential in busy clinical settings

## Discussion

The COVID-19 pandemic created a rapid and unprecedented disruption to the medical education system nationwide, and particularly impacted medical schools in New York City. The clerkship year in medical school has always centered around care of patients. The inability for students to enter the physical clinical environment challenged the core aspect of their clinical training. In response to this challenge, we have demonstrated the successful real-time development and implementation of a program of telehealth experiences for medical students in a healthcare system strained by the pandemic. Importantly, this program ensured student safety but still allowed them to participate in a meaningful and educational way in the care of patients and continue their academic progress.

Prior to the COVID-19 pandemic, there has been ongoing growth in the use of telehealth models in the practice of medicine, and medical student training in telehealth has been increasing over the years^5^. Previously, the majority of the training at our institution focused on the mechanics and logistics of telehealth encounters and use of standardized patients for simulated telehealth encounters. Real-time development and implementation of a telehealth program for medical students with more applied application of telehealth skills was borne out of necessity due to COVID-19 and can inform processes at other schools. Even after the COVID-19 crisis has passed, we anticipate that aspects of these interactive telehealth experiences will be formalized into our core clinical curriculum. Medical students will need more structured education in communication techniques, history taking and physical exam strategies and disposition planning done over phone and video-based technology as telehealth becomes part of the ‘new normal’ in health care delivery.

Despite the increasing use of telehealth modalities in patient care, and the successful development of the RITE program at WCM, the authors agree that these types of experiences will not or should not replace student experiences in the physical clinical environment. In fact, the RITE experiences were designed to provide students with elective credit, but not academic credit towards clerkship completion. Telehealth education should complement, but not replace, in-person clinical training. As a next step, we will consider whether we should develop a longitudinally integrated telehealth experience as a component of each clerkship, or a separate stand-alone telehealth program for senior medical students.
